# Short- and Long-Term Outcomes of Pancreatic Cancer Resection in Elderly Patients: A Nationwide Analysis

**DOI:** 10.1245/s10434-022-11831-7

**Published:** 2022-06-02

**Authors:** Anne Claire Henry, Thijs J. Schouten, Lois A. Daamen, Marieke S. Walma, Peter Noordzij, Geert A. Cirkel, Maartje Los, Marc G. Besselink, Olivier R. Busch, Bert A. Bonsing, Koop Bosscha, Ronald M. van Dam, Sebastiaan Festen, Bas Groot Koerkamp, Erwin van der Harst, Ignace H. J. T. de Hingh, Geert Kazemier, Mike S. Liem, Vincent E. de Meijer, Vincent B. Nieuwenhuijs, Daphne Roos, Jennifer M. J. Schreinemakers, Martijn W. J. Stommel, I. Quintus Molenaar, Hjalmar C. van Santvoort, for the Dutch Pancreatic Cancer Group

**Affiliations:** 1 Department of Surgery, Regional Academic Cancer Center Utrecht, St. Antonius Hospital Nieuwegein, University Medical Center Utrecht, Utrecht University, Utrecht, The Netherlands; 2 Department of Anesthesiology and Intensive Care, St. Antonius Hospital Nieuwegein, Utrecht, The Netherlands; 3 Department of Medical Oncology, Regional Academic Cancer Center Utrecht, Meander Medical Center Amersfoort, University Medical Center Utrecht, Utrecht, The Netherlands; 4 Department of Medical Oncology, Regional Academic Cancer Center Utrecht, St. Antonius Hospital Nieuwegein, University Medical Center Utrecht, Utrecht, The Netherlands; 5 Department of Surgery, Cancer Center Amsterdam, Amsterdam UMC, University of Amsterdam, Amsterdam, The Netherlands; 6 Department of Surgery, Leiden University Medical Center, Leiden, The Netherlands; 7 Department of Surgery, Jeroen Bosch Hospital, Den Bosch, The Netherlands; 8 Department of Surgery, Maastricht UMC+, Maastricht, The Netherlands; 9 Department of Surgery, Onze Lieve Vrouwen Gasthuis, Amsterdam, The Netherlands; 10 Department of Surgery, Erasmus MC, Rotterdam, The Netherlands; 11 Department of Surgery, Maasstad Hospital, Rotterdam, The Netherlands; 12 Department of Surgery, Catharina Hospital, Eindhoven, The Netherlands; 13 Department of Surgery, Cancer Center Amsterdam, Amsterdam UMC, Vrije Universiteit, Amsterdam, The Netherlands; 14 Department of Surgery, Medical Spectrum Twente, Enschede, The Netherlands; 15 Department of Surgery, University of Groningen and University Medical Center Groningen, Groningen, The Netherlands; 16 Department of Surgery, Isala, Zwolle, Zwolle, The Netherlands; 17 Department of Surgery, Reinier de Graaf Group, Delft, The Netherlands; 18 Department of Surgery, Amphia Hospital, Breda, The Netherlands; 19 Department of Surgery, Radboud University Medical Center, Nijmegen, The Netherlands

## Abstract

**Background:**

The number of elderly patients with pancreatic cancer is growing, however clinical data on the short-term outcomes, rate of adjuvant chemotherapy, and survival in these patients are limited and we therefore performed a nationwide analysis.

**Methods:**

Data from the prospective Dutch Pancreatic Cancer Audit were analyzed, including all patients undergoing pancreatic cancer resection between January 2014 and December 2016. Patients were classified into two age groups: <75 and ≥75 years. Major complications (Clavien–Dindo grade 3 or higher), 90-day mortality, rates of adjuvant chemotherapy, and survival were compared between age groups. Factors associated with start of adjuvant chemotherapy and survival were evaluated with logistic regression and multivariable Cox regression analysis.

**Results:**

Of 836 patients, 198 were aged ≥75 years (24%) and 638 were aged <75 years (76%). Median follow-up was 38 months (interquartile range [IQR] 31–47). Major complications (31% vs. 28%; *p* = 0.43) and 90-day mortality (8% vs. 5%; *p* = 0.18) did not differ. Adjuvant chemotherapy was started in 37% of patients aged ≥75 years versus 69% of patients aged <75 years (*p* < 0.001). Median overall survival (OS) was 15 months (95% confidence interval [CI] 14–18) versus 21 months (95% CI 19–24; *p* < 0.001). Age ≥75 years was not independently associated with OS (hazard ratio 0.96, 95% CI 0.79–1.17; *p* = 0.71), but was associated with a lower rate of adjuvant chemotherapy (odds ratio 0.27, 95% CI 0.18–0.40; *p* < 0.001).

**Conclusions:**

The rate of major complications and 90-day mortality after pancreatic resection did not differ between elderly and younger patients; however, elderly patients were less often treated with adjuvant chemotherapy and their OS was shorter.

**Supplementary Information:**

The online version contains supplementary material available at 10.1245/s10434-022-11831-7.

Pancreatic cancer is a devastating disease, frequently affecting older people. In 2019, nearly half of all patients with pancreatic ductal adenocarcinoma (PDAC) in The Netherlands were aged ≥75 years at the time of diagnosis.^1^ Due to aging of the population, combined with an increased life expectancy in industrial countries, the number of elderly patients with pancreatic cancer is growing.^2^

Optimal treatment for pancreatic cancer consists of surgical resection in combination with chemotherapy;^3–5^ however, pancreatic surgery is associated with high postoperative complication rates.^6,7^ Although it is commonly felt that age alone should not be a contraindication for resection of pancreatic cancer, surgeons are generally hesitant to perform these major surgical procedures in the elderly.^8–13^ Previous studies have suggested that older patients have a higher risk of major postoperative complications due to comorbid conditions and functional impairment.^3,14–18^ It has also been shown that older patients may be less likely to receive adjuvant chemotherapy due to frailty, even though chemotherapy is associated with improved survival.^19–21^ However, the independent impact of age on clinical outcomes remains controversial.^8,22–27^ Furthermore, most previous studies on pancreatic surgery in the elderly were performed in selected patients from single-center studies with small study populations and without correction for frailty.^8,14,15,19,22,24–26,28–31^ A reflection of daily clinical practice in terms of short- and long-term outcomes of elderly patients after resection for pancreatic cancer on a nationwide scale is lacking.

In The Netherlands, efforts have been made to improve outcomes after pancreatic cancer resection. In 2013, a nationwide clinical audit—the Dutch Pancreatic Cancer Audit (DPCA)—was established for quality assessment of perioperative care in pancreatic surgery.^32^ Over the last decade, pancreatic surgery has been centralized and regional partnerships have emerged. It is stated that patients benefit from centralization due to increased resection rates and reduced morbidity rates.^3,33–35^ Furthermore, multidisciplinary team meetings have been initiated to carefully screen patients on frailty and surgical risk, aiming to improve selection of patients for optimal treatment, while also paying attention to prehabilitation in order to get patients fit for surgery.^36,37^ It is likely that these improvements have also benefited elderly patients with pancreatic cancer. We therefore performed the current study with the aim to investigate short-term outcomes, the rate of adjuvant chemotherapy, and survival in elderly patients undergoing pancreatic cancer resection in a recent nationwide cohort in The Netherlands.

## Methods

### Study Design

This was a post hoc analysis of the DPCA prospective database. All patients undergoing resection for histologically proven PDAC between January 2014 and December 2016 in all 17 centers collaborating in the Dutch Pancreatic Cancer Group were included,^38,39^ including patients with resectable and borderline resectable PDAC. There were no exclusion criteria. We adhered to the Strengthening the Reporting of Observational studies in Epidemiology (STROBE) guidelines.^40^

### Data Collection

Prospective baseline and perioperative data were retrieved from the available prospective database. Data on ethnicity were not included in the database. Additional data on frailty characteristics, follow-up, treatment, and survival were collected retrospectively from hospital records. The Charlson Comorbidity Index (CCI) was calculated using the MDCalc CCI calculator,^41^ and TNM status was assessed according to the 8th Edition of the American Joint Committee on Cancer (AJCC) guidelines.^42^ Resection margins were considered positive if tumor cells were present within 1 mm of the resection margins, apart from the anterior surface.^43^ Frailty characteristics consisted of polypharmacy (use of five or more medicaments at the time of diagnosis),^44^ preoperative anemia (female hemoglobin <7.4 mmol/L, male hemoglobin <8.1 mmol/L),^45^ decreased renal function,^46,47^ CCI ≥2^44^, body mass index (BMI) <18.5 or ≥31,^23^ and American Society of Anesthesiologists (ASA) score ≥3.^44,48,49^ A decreased renal function was, in terms of the preoperative estimated glomerular filtration rate (eGFR), defined as mild (60–89 mL/min/1.73 m^2^), mild to moderate (45–59 mL/min/1.73 m^2^), moderate to severe 30–44 mL/min/1.73 m^2^, or severe 15–29 mL/min/1.73 m^2^.^46,47^

### Outcomes

Outcomes of interest were major complications, intensive care unit (ICU) readmission, 90-day mortality, rate of adjuvant chemotherapy (completion of at least one cycle), recurrence rate, disease-free survival (DFS), and overall survival (OS). Major complications were defined as Clavien–Dindo grade 3 or higher.^50^ PDAC recurrence had to be either pathologically proven or suspected through cross-sectional imaging, preferably confirmed by consensus at a multidisciplinary meeting. DFS was defined as the time from the date of resection to the date of diagnosis of PDAC recurrence, while OS was defined as the time from the date of surgery to either death from any cause or last follow-up. If survival data were missing, patients were censored at the date of last follow-up.

### Statistical Analyses

Missing data were considered missing at random and were therefore managed by multiple imputation according to a Markov chain Monte Carlo method (5 imputations, 10 iterations).^51^ Parametric continuous variables were reported as mean with standard deviation (SD) and compared using the Student’s *t*-test; non-parametric continuous variables were presented as median with interquartile range (IQR) and compared using the Mann-Whitney U-test; and categorical variables were reported as frequencies and compared using the Chi-square test.

Patients were divided into two age groups: <75 and ≥75 years. In the DPCA, the median age of patients with PDAC in The Netherlands is 68 years, with the population aging over time.^1,52^ Therefore, this study defined patients aged over 75 years as the true elderly. Univariate analysis was performed to compare major complications, ICU readmission, and 90-day mortality between both age groups. OS and DFS were evaluated using the Kaplan–Meier analysis, compared using the log-rank test, and presented as median with 95% confidence intervals (CIs). To minimize the influence of postoperative mortality on the results of long-term survival, OS and DFS were also assessed in patients without 90-day mortality. The influence of age ≥ 75 years on OS was assessed using multivariable Cox proportional hazard analyses and reported as hazard ratios (HRs) with 95% CIs, adjusted for potential confounders. These included sex, BMI, preoperative serum carbohydrate antigen (CA) 19–9, frailty characteristics, location and size of the tumor, microscopic perineural invasion, tumor differentiation, and number of positive lymph nodes. Stratified analyses were performed for patients who received adjuvant chemotherapy and patients who did not. Multivariable logistic regression analyses, adjusted for potential confounders, were performed to assess the association between age ≥ 75 years and administration and completion of adjuvant chemotherapy. A sensitivity analysis for patients aged ≥ 80 years was also performed. Results are given as odds ratios (ORs) with 95% CIs. A two-tailed *p*-value < 0.05 was considered to indicate statistical significance. Statistics were performed using R version 1.3.1093 (Bell Laboratories, Windsor, NH, USA) with the ‘survival’, ‘ggplot’, and ‘mice’ packages.

## Results

A total of 836 patients were included (electronic supplementary material [ESM] Appendix Table 1), of whom 638 (76%) were aged < 75 years and 198 (24%) were aged ≥ 75 years. In patients aged < 75 years, the median age was 66 years (IQR 58–70), and in patients aged ≥ 75 years, the median age was 78 years (IQR 76–80). Patient and tumor characteristics of both groups are summarized in Table 1. The median overall follow-up period was 38 months (IQR 31–47).

**Table 1:** Patient, tumor, and treatment characteristics of 638 patients aged <75 years and 198 patients aged ≥75 years after resection for pancreatic cancer

	Age < 75 years [*N* = 638]	Age ≥ 75 years [*N* = 198]	*p*-value^a^
Male sex	353 (55)	106 (54)	0.72
BMI < 18.5 or ≥ 31	74 (12)	17 (9)	0.28
Charlson Comorbidity Index < 2 ≥ 2	390 (61) 248 (39)	82 (41) 116 (59)	<0.001
ASA classification I–II III–IV	512 (80) 126 (20)	131 (66) 67 (34)	<0.001
ECOG performance score at primary diagnosis 0–1 2–4	563 (88) 75 (12)	164 (83) 34 (17)	0.05
Preoperative serum log CA19-9 [median (IQR)]	120 (30–480)	151 (29–539)	0.09
Preoperative bilirubin, µmol/L [median (IQR)]	24 (9–89)	23 (9–75)	0.91
Preoperative eGFR, mL/min/1.73 m^2^ Normal (> 90) Mildly decreased (60–89) Mildly to moderately decreased (45–59) Moderately to severely decreased (30–45) Severely decreased (<30)	193 (30) 337 (53) 73 (11) 32 (5) 3 (0)	41 (21) 125 (63) 20 (10) 10 (5) 3 (1)	0.03
Preoperative anemia	335 (52)	106 (53)	0.88
Number of medicaments < 5 ≥ 5	403 (63) 235 (37)	107 (54) 91 (46)	0.03
Neoadjuvant chemotherapy	53 (8)	9 (5)	0.11
Method of surgery Open Laparoscopic Robot	582 (91) 51 (8) 5 (1)	175 (88) 22 (11) 1 (1)	0.38
Type of resection Pancreatoduodenectomy Distal pancreatectomy Total pancreatectomy	523 (82) 89 (14) 26 (4)	159 (80) 31 (16) 8 (4)	0.83
Tumor location Head Body/tail	543 (85) 95 (15)	166 (84) 32 (16)	0.75
Vascular resection	175 (27)	53 (27)	0.88
Microscopic perineural invasion	554 (87)	172 (87)	0.99
Microscopic lymphovascular invasion	421 (66)	122 (62)	0.31
Tumor size, cm^b^ [mean ± SD]	3.2 ± 1.3	3.2 ± 1.1	0.75
Tumor differentiation Well/moderate Poor	433 (68) 204 (32)	130 (71) 58 (29)	0.32
Total number of resected lymph nodes [median (IQR)]	16 (11–21)	12 (9–18)	<0.001
Number of positive lymph nodes [median (IQR)]	2 (0–4)	2 (0–4)	0.57
TNM stage, AJCC 7th edition ≤ Stage 2a ≥ Stage 2b	62 (10) 576 (90)	16 (8) 182 (92)	0.53
Resection margin status R0 >1.0 mm R1 ≤ 1.0 mm	325 (51) 313 (49)	86 (44) 112 (56)	0.08

Data are expressed as *n* (%) unless otherwise specified

Percentages may not sum to 100 because of rounding

^a^The data were statistically analyzed between both groups using the Chi-square test for categorical variables and Fisher’s exact test when groups consisted of fewer than five patients. The *t*-test was used for normally distributed continuous variables, and the Wilcoxon rank test was used for non-normally distributed continuous variables

^b^Maximum diameter of the tumor

*SD* standard deviation, *BMI* body mass index, *ASA* American Society of Anesthesiologists, *ECOG* Eastern Cooperative Oncology Group, *CA19-9* carbohydrate antigen 19-9, *IQR* interquartile range, *eGFR* estimated glomerular infiltration rate, *AJCC* American Joint Committee on Cancer

### Short-Term Outcomes

In 62 patients (31%) aged ≥ 75 years and 179 patients (28%) aged < 75 years, one or more major complications occurred (*p* = 0.43) [Table 2]. Readmission to the ICU was necessary for 40 patients (20%) versus 82 patients (13%) [*p* = 0.01]. In addition, 90-day mortality occurred in 16 patients (8%) versus 33 patients (5%) [*p* = 0.18]. In a post hoc multivariable logistic regression analysis adjusted for frailty, sex, BMI, and location and size of the tumor, age (< 75 years vs. ≥ 75 years) was also not associated with major complications or 90-day mortality (OR 1.07, 95% CI 0.74–1.53, *p* = 0.72; and 1.39, 95% CI 0.72–2.69, *p* = 0.32, respectively).

**Table 2:** Univariate analysis of short- and long-term outcomes of 638 patients aged <75 years and 198 patients aged ≥75 years after resection for pancreatic cancer

	Age < 75 years [*N* = 638]	Age ≥ 75 years [*N* = 198]	*p*-value^a^
Major complications	179 (28)	62 (31)	0.43
Length of hospital stay, days [median (IQR)]	11 (8–15)	14 (9–20)	< 0.001
Adjuvant chemotherapy^c^	429 (69)	71 (37)	< 0.001
Type of adjuvant chemotherapy^b,d^ Gemcitabine monotherapy FOLFIRINOX Other Unknown	408 (95) 5 (1) 2 (0) 10 (2)	66 (93) 1 (1) 1 (1) 0 (0)	< 0.001
No. of cycli of adjuvant chemotherapy [median (IQR)]^b,e^	6 (4–6)	6 (3–6)	0.009
≥80% of prescribed cycles completed^b,e^	288 (73)	36 (64)	0.23
90-day mortality	33 (5)	16 (8)	0.18
Overall survival, months [median (95% CI)]	21 (19–24)	15 (14–18)	< 0.001
Disease-free survival, [median (95% CI)]^f^	16 (14–17)	12 (10–14)	< 0.001
Recurrence^f^	435 (81)	122 (81)	0.99

Data are expressed as *n* (%) unless otherwise specified

Percentages may not sum to 100 because of rounding

*IQR* nterquartile range, *FOLFIRINOX* 5-fluorouracil, leucovorin, irinotecan, oxaliplatin chemotherapy, *CI* confidence interval

^a^The data were statistically analyzed between both groups using the Chi-square test for categorical variables and Fisher’s exact test when groups consisted of fewer than five patients. The *t*-test was used for normally distributed continuous variables, and the Wilcoxon rank test was used for non-normally distributed continuous variables

^b^Calculated in a subset of patients who started with adjuvant chemotherapy (429 patients aged < 75 years vs. 71 patients aged ≥ 75 years)

^c^28 missing

^d^7 missing

^e^49 missing

^f^145 missing

### Adjuvant Chemotherapy

Adjuvant chemotherapy was started in 71 patients aged ≥ 75 years (37%) versus 429 patients aged < 75 years (69%) [*p* < 0.001]. Once chemotherapy had commenced, ≥80% of the prescribed cycles were completed in 36 (64%) versus 288 (73%) patients (*p* = 0.23) [Table 2]. Multivariable analysis showed that age ≥ 75 years was independently associated with start of adjuvant chemotherapy (OR 0.27, 95% CI 0.18–0.40; *p* < 0.001) [Table 3]. Furthermore, adjuvant chemotherapy was less often administered to patients with a CCI score ≥ 2 (OR 0.62, 95% CI 0.43–0.90; *p* = 0.01) or major complications (OR 0.21, 95% CI 0.15–0.30; *p* < 0.001). With regard to frailty, significantly more elderly who did not receive adjuvant chemotherapy had high CCI (≥ 2) and ASA (≥ 3) scores compared with elderly who did receive adjuvant chemotherapy (68 vs. 44%, and 41 vs. 20%, respectively) [ESM Appendix Table 2]. Once started with adjuvant chemotherapy, age ≥ 75 years was not significantly associated with the completion of ≥ 80% of the prescribed cycles (OR 0.69, 95% CI 0.37–1.29; *p* = 0.24) [Table 3]. Stratified Kaplan–Meier curves for older versus younger patients who started with adjuvant chemotherapy showed an OS of 25 months (95% CI 18–37) and 28 months (95% CI 25–31) [*p *= 0.18], respectively, and a DFS of 17 months (95% CI 13–25) and 19 months (95% CI 16–21) [*p* = 0.069], respectively (Figs. 3 and 4).

**Table 3:** Multivariable logistic regression analysis to assess the independent impact of age ≥75 years on start and completion ≥80% of adjuvant chemotherapy in 836 patients after resection of pancreatic cancer

	Start adjuvant chemotherapy			Completion ≥80% of adjuvant chemotherapy		
	OR	95% CI	*p*-value	OR	95% CI	*p*-value
Age (≥75 vs. <75 years)	0.27	0.18–0.40	< 0.001	0.69	0.37–1.29	0.24
Sex (male vs. female)	0.32	0.93–1.87	0.12	0.65	0.40–1.06	0.09
Charlson Comorbidity Index (≥2 vs. <2)	0.62	0.43–0.90	0.01	0.68	0.42–1.11	0.12
Polypharmacia (≥5 vs. <5 medicaments)	0.79	0.55–1.14	0.21	1.16	0.70–1.92	0.56
Anemia (yes vs. no)	0.88	0.53–1.47	0.63	1.13	0.58–2.18	0.73
BMI (<18.5 or ≥31 vs. 18.5–31)	1.28	0.75–2.20	0.37	0.75	0.38–1.48	0.41
Renal dysfunction, eGFR (mL/min/1.73 m^2^) Mildly decreased (60–89) Mildly to moderately decreased (45–59) Moderately to severely decreased (30–45) Severely decreased (<30)	0.99 0.78 0.90 1.04	0.61–1.63 0.39–1.58 0.28–2.83 0.04–24.66	0.98 0.49 0.86 0.98	0.73 0.85 1.13 –	0.39–1.35 0.33–2.20 0.12–10.29 –	0.33 0.74 0.91 0.99
Major complications (yes vs. no)	0.21	0.15–0.30	< 0.001	1.80	0.98–3.30	0.06
Location tumor (body/tail vs. head)	0.84	0.53–1.32	0.45	1.44	0.71–2.90	0.31
Tumor size	0.96	0.83–1.10	0.52	0.89	0.74–1.08	0.24
Tumor differentiation (poor vs. well/moderate)	0.78	0.53–1.15	0.22	0.99	0.60–1.63	0.98
Preoperative log CA19-9	0.98	0.89–1.08	0.67	0.97	0.86–1.11	0.69
Positive resected lymph nodes	1.00	0.95–1.05	0.92	1.00	0.93–1.08	0.93
Resection margin status (R1 vs. R0)	0.71	0.50–1.01	0.05	1.53	0.97–2.41	0.07
Neural invasion (yes vs. no)	0.94	0.50–1.74	0.84	0.98	0.50–1.91	0.95

*OR* odds ratio, *CI* confidence interval, *BMI* body mass index, *eGFR* estimated glomerular filtration rate, *CA19-9* carbohydrate antigen 19-9

### Disease Recurrence and Survival

**Fig. 1:**
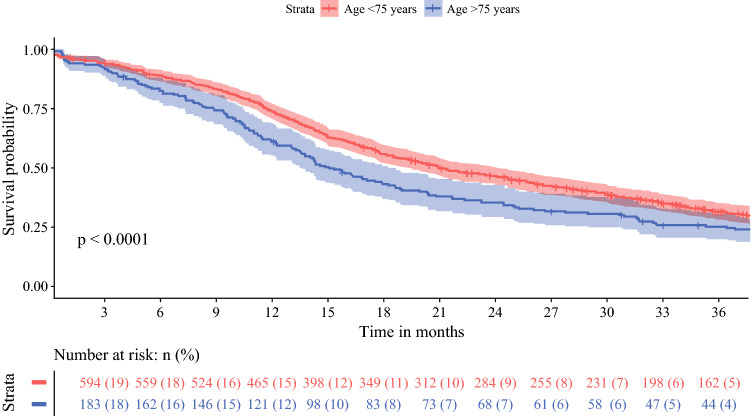
Overall survival of 638 patients aged < 75 years and 198 patients aged ≥ 75 years after resection for pancreatic cancer

**Fig. 2:**
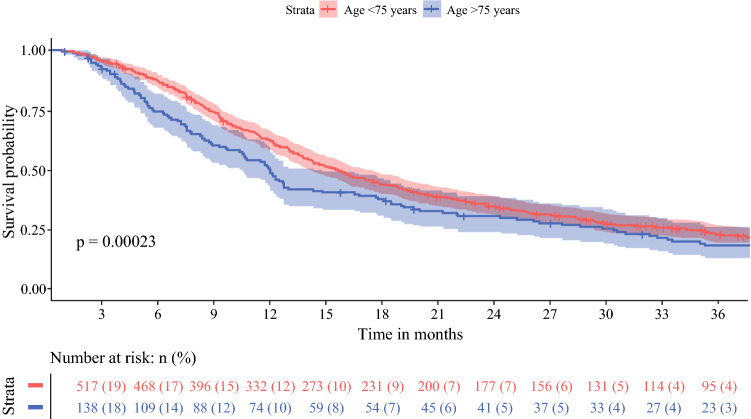
Disease-free survival of 638 patients aged < 75 years and 198 patients aged ≥ 75 years after resection for pancreatic cancer

Both age groups developed recurrence in 81% of patients (122 patients ≥75 years of age versus 435 patients <75 years of age; *p* = 0.99) [Table 2]. However, the median DFS was 12 months (95% CI 10–14 months) for patients aged ≥75 years versus 16 months (95% CI 14–17 months) for patients aged <75 years (*p* < 0.001). OS was 15 months (95% CI 14–18 months) and 21 months (95% CI 19–24 months) for patients aged ≥75 and <75 years, respectively (*p* < 0.001) [Figs. 1 and 2].

In the analysis excluding patients with 90-day mortality, the DFS was 12 months (95% CI 11–14 months) for patients aged ≥ 75 years and 16 months (95% CI 14–17 months) for patients aged < 75 years (*p* < 0.001), while the OS was 17 months (95% CI 14–20 months) and 23 months (95% CI 21–26 months) for older and younger patients, respectively (*p* < 0.001).

When adjusted for potential confounders, including frailty characteristics, age ≥ 75 years was not independently associated with either DFS (HR 0.99, 95% CI 0.79–1.23; *p* = 0.90) or OS (HR 0.96, 95% CI 0.79–1.17; *p* = 0.71) [Table 4]. Major complications were associated with lower OS (HR 1.39, 95% CI 1.17–1.66; *p* < 0.001). Certain tumor characteristics (tumor size, tumor differentiation, serum CA19-9, number of resected (positive) lymph nodes, resection margin status, and neural invasion) were also significantly associated with lower DFS and OS (Table 4). Start of adjuvant chemotherapy was associated with improved DFS (HR 0.56, 95% CI 0.46–0.68; *p *< 0.001) and OS (HR 0.45, 95% CI 0.37–0.53).

**Fig. 3:**
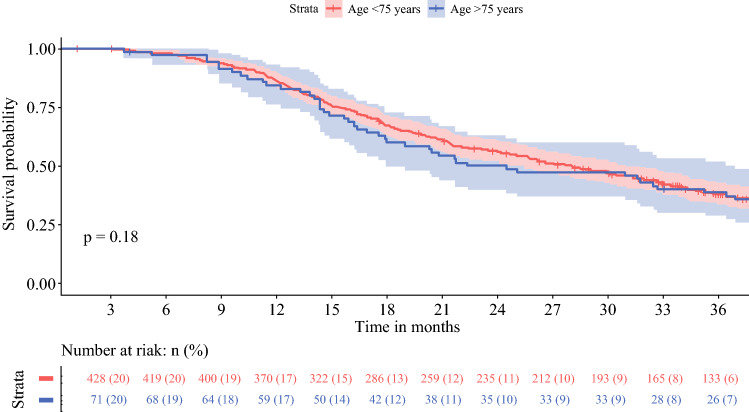
Overall survival in 71 patients aged ≥ 75 years and in 429 patients aged < 75 years after resection for adjuvant chemotherapy

**Fig. 4:**
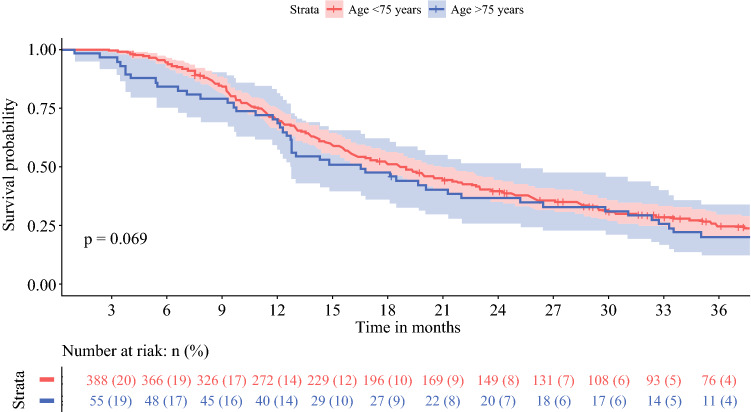
Disease-free survival in 71 patients aged ≥ 75 years and in 429 patients aged < 75 years after resection for adjuvant chemotherapy

**Table 4:** Multivariable Cox regression analysis to assess the independent impact of age ≥ 75 years on overall survival and disease-free survival in 836 patients after resection of pancreatic cancer

	Overall survival			Disease-free survival		
	HR	95% CI	*p*-value	HR	95% CI	*p*-value
Age (≥ 75 vs. < 75 years)	0.96	0.79–1.17	0.71	0.99	0.79–1.23	0.90
Sex (male vs. female)	0.95	0.80–1.12	0.51	0.93	0.78–1.12	0.45
Charlson Comorbidity Index (≥ 2 vs. < 2)	1.10	0.91–1.32	0.33	1.10	0.90–1.33	0.36
Polypharmacia (≥ 5 vs. < 5 medicaments)	0.94	0.78–1.13	0.49	0.92	0.75–1.12	0.40
Anemia (yes vs. no)	1.13	0.96–1.34	0.15	0.99	0.83–1.19	0.95
BMI (< 18.5 or ≥31 vs. 18.5–31)	1.10	0.85–1.43	0.48	0.99	0.75–1.30	0.92
Renal dysfunction, eGFR (mL/min/1.73 m^2^) Mildly decreased (60–89) Mildly to moderately decreased (45–59) Moderately to severely decreased (30–45) Severely decreased (< 30)	1.01 0.94 0.92 1.91	0.84–1.21 0.71–1.26 0.62–1.35 0.74–4.96	0.93 0.69 0.66 0.18	1.01 0.90 0.89 0.94	0.83–1.22 0.67–1.23 0.59–1.35 0.25–3.60	0.95 0.52 0.59 0.93
Major complications (yes vs. no)	1.39	1.17–1.66	< 0.001	1.08	0.89–1.31	0.46
Location tumor (body/tail vs. head)	0.98	0.77–1.24	0.87	0.93	0.72–1.20	0.57
Tumor size	1.13	1.06–1.21	< 0.001	1.14	1.06–1.22	< 0.001
Tumor differentiation (poor vs. well/moderate)	1.39	1.16–1.65	< 0.001	1.40	1.16–1.68	< 0.001
Preoperative log CA19-9	1.05	1.01–1.09	0.03	1.07	1.02–1.11	< 0.01
Positive resected lymph nodes	1.06	1.04–1.09	< 0.001	1.08	1.05–1.11	< 0.001
Resection margin status (R1 vs. R0)	1.27	1.08–1.50	< 0.01	1.34	1.13–1.60	< 0.001
Neural invasion (yes vs. no)	1.71	1.29–2.26	< 0.001	1.60	1.20–2.13	< 0.01
Adjuvant chemotherapy (yes vs. no)	0.45	0.37–0.53	< 0.001	0.56	0.46–0.68	< 0.001

*HR* hazard ratio, *CI* confidence interval, *BMI* body mass index, *eGFR* estimated glomerular filtration rate

### Sensitivity Analysis Octogenerians

In addition, a sensitivity analysis of all patients aged ≥80 years was performed. Results of the univariate analysis of 53 patients aged ≥80 years versus 783 patients aged <80 years are presented in ESM Appendix Table 3. In 16 patients (30%) aged ≥80 years, one of more major complications occurred compared with 225 patients (29%) aged < 80 years (*p* = 0.94). Ninety-day mortality was 13% (*n* = 7) in the older patients compared with 5% (*n* = 42) in the younger patients (*p* = 0.03). In the multivariable logistic regression analysis, assessed for frailty, age was not independently associated with 90-day complication-related mortality (ESM Appendix Table 4). Adjuvant chemotherapy was started in 5 patients (10%) aged ≥80 years and 495 patients (65%) aged <80 years (*p* < 0.001). None of the older patients completed ≥ 80% of the prescribed cycles. Patients aged ≥80 years showed a median DFS of 10 months (95% CI 7–20 months) compared with 15 months (95% CI 14–16 months) in the younger patients (*p* < 0.01). Median OS was 14 months (95% CI 12–20 months) and 20 months (95% CI 18–22 months) [*p* < 0.001], respectively.

## Discussion

This nationwide study found that short-term outcomes after pancreatic resection, including the incidence of major complications and 90-day mortality, were not significantly different for patients aged ≥ 75 years compared with younger patients; however, long-term survival was shorter in the elderly patients. It was also observed that elderly less often received adjuvant chemotherapy after resection for pancreatic cancer.

The evidence for beneficial outcomes after resection for elderly patients with pancreatic cancer is not straightforward. Most of the studies reporting on elderly are performed in selected patients from single-center studies with small study groups, and the representativeness of these outcomes in a general population can be questioned.^8,14,15,19,22,24–26,28–31^ Furthermore, the direct impact of age as an independent predictor for short-term postsurgical outcomes remains controversial.^8,22–27^ In the current study, the rates of major complications and 90-day mortality were lower compared with previous studies, and not significantly different between both age groups.^8,14,15,19,24,25,29–31^ It appears that nowadays the risks of performing pancreatic cancer surgery in patients aged ≥ 75 years are comparable with that of younger patients, also on a nationwide scale.

Equally important, the impact of age on long-term oncological outcomes after resection of pancreatic cancer remains ambiguous. Previous studies on OS in elderly reported no statistically significant difference between older and younger patients (median 9–26 vs. 12–24 months), and age was not independently associated with OS in multivariable analysis.^8, 15,19,24,31^ Moreover, DFS was comparable between both age groups (median 7–13 vs. 15 months).^19,53^ Our results show significant differences in median OS and DFS for patients aged ≥ 75 years when compared with patients aged <75 years, i.e. 15 versus 21 months (*p *< 0.001) and 12 versus 16 months (*p* < 0.001). This difference might be explained by the fact that consecutive patients were included, as the nationwide registry is obligatory and has been validated so as to not miss any cases. Consequently, the risk of selection bias in this study has been decreased. Moreover, the relatively low rate of adjuvant chemotherapy in elderly that was observed in this cohort might explain the shorter long-term survival. In terms of frailty, this could be explained by the worse CCI and ASA scores in elderly patients who did not receive chemotherapy in the adjuvant setting. However, more importantly, it should be considered that the decision whether or not to start with adjuvant chemotherapy in elderly is made differently with regard to their life expectancy compared with younger patients. In this perspective, not all elderly patients may want to undergo adjuvant treatment. On the other hand, in elderly who did receive adjuvant chemotherapy, OS and DFS were comparable with that in younger patients who also received adjuvant chemotherapy. Most patients in this cohort were treated with adjuvant gemcitabine. With current 5-fluorouracil, leucovorin, irinotecan, oxaliplatin (FOLFIRINOX) chemotherapy regimens, survival outcomes are improving;^54^ however, FOLFIRINOX is associated with considerable more toxicity than gemcitabine.^54^ For frail elderly patients, reduced-dose chemotherapy or the use of modified FOLFIRINOX could provide a solution.^55,56^ To increase the potential for receiving chemotherapeutical regimens, a neoadjuvant chemotherapy approach could also be a suitable alternative for this patient group, given that they were considered to be fit enough for pancreatic resection.^57–59^

Previous studies have reported that frailty is a prognostic factor for postoperative morbidity and mortality after major abdominal surgery.^60,61^ After pancreatic resection, it has also been shown that frailty is associated with an increased incidence of major complications and death, as well as worse survival outcomes.^36,49,62,63^ Several validated scoring systems can be used to assess frailty, based on a multidimensional approach of physical, mental, and social status.^64,65^ In this study, we used frailty characteristics derived from the physical domain, since preoperative mental and social assessments were not yet standardized in clinical practice during the study period.^23,44–49^ Evaluation of outcomes with regard to a complete preoperative frailty assessment could be the focus for future research.

The analysis presented in this study, using a multicenter, nationwide prospective patient cohort, provides valuable insights into the short- and long-term outcomes of elderly after pancreatic resection in a recent real-world patient population. The inclusion period (2014–2016) comprises a period in which nationwide improvements in perioperative care for pancreatic cancer patients have been implemented in The Netherlands. For less physically fit patients, prehabilitation, i.e. preoperative exercise training to optimize functional deficits, nutritional interventions, psychological support, and coaching towards lifestyle changes, has been shown to reduce the risk of postoperative morbidity and is more frequently applied.^36,37^ Furthermore, it has been suggested that treatment at specialized centers and accounting for comorbidities in the decision-making process leads to improved outcomes for elderly.^14,22,23,25^ A previous Dutch study demonstrated an increase in pancreatic resections among elderly patients (≥ 75 years) over a time period between 2005 and 2013, resulting in decreased postoperative mortality in high-volume hospitals.^33^ Therefore, this study concluded that elderly could benefit from centralization when undergoing pancreatic resection.

This study has some limitations. First, data regarding elderly patients with resectable PDAC who did not undergo a resection, including insights into the decision-making process, were not available. Second, although a prospective database was used for baseline and perioperative data, data on follow-up and recurrence treatment were collected retrospectively; hence, only objective characteristics on physical frailty could be collected. It was not possible to obtain detailed information on mental and social frailty scores because this was not always reported in the electronic patient files. Nevertheless, in contrast to most studies, we did adjust for the frailty characteristics that were available. Third, the definition of true elderly remains uncertain. In this study, patients aged 75 years or older were assumed as the true elderly, however, different cut-offs in age have been suggested.^66^ Some studies propose octogenerians (aged ≥ 80 years) as the true elderly. We therefore performed a sensitivity analysis in octogenerians and the results were in line with the main analyses. Although the rate of postoperative complications did not differ, there was a marked increase in 90-day mortality for patients aged ≥ 80 years. In multivariable logistic regression analysis, age was not independently associated with 90-day complication-related mortality when assessed for frailty. This suggests that failure to rescue rates may be increased in octogenerians due to frailty, and caution should be maintained in the decision making on resection in these patients. Fourth, considerations in the shared decision-making process that led to either the start or omission of adjuvant chemotherapy could not always be identified accurately. This could have given more insights into the reasons to refrain from adjuvant treatment in elderly.

## Conclusion

Following recent advancements in pancreatic cancer care, short-term outcomes after resection for pancreatic cancer did not differ between older and younger patients; however, only one-third of the elderly received adjuvant chemotherapy. Survival was shorter in elderly patients. These real-life data from a nationwide, multicenter cohort provide new insights for future shared-decision making on surgery and adjuvant chemotherapy for pancreatic cancer in elderly patients.

## Supplementary Information

Below is the link to the electronic supplementary material.

Supplementary file1 (DOCX 39 kb)
