# Supplementary material for: Short- and Long-Term Outcomes of Pancreatic Cancer Resection in Elderly Patients: A Nationwide Analysis
Source: Ann Surg Oncol. 2022 Jun 2;29(9):6031–42. doi: 10.1245/s10434-022-11831-7 (PMC9356963; doi:10.1245/s10434-022-11831-7)
Supplement: Supplementary file 1 — Supplementary file1 (DOCX 39 kb) [file 10434_2022_11831_MOESM1_ESM.docx]

**Short- and long-term outcomes of resection of pancreatic cancer in elderly patients: a nationwide analysis**

***Appendix***

| **Appendix Table 1. Patient, tumor and treatment characteristics of 836 patients after resection for pancreatic cancer** | | | |
| --- | --- | --- | --- |
|  | **Original cohort**  **n = 836** | **Missing data**  **n (%)** | **After imputation**  **n = 836** |
| Age (years), mean ± SD | 67 ± 9 | - | 67 ± 9 |
| Age (years), n (%)  <65  65-70  70-75  75-80  80-85  >85 | 306 (37)  171 (20)  161 (19)  145 (17)  48 (6)  5 (1) | - | 306 (37)  171 (20)  161 (19)  145 (17)  48 (6)  5 (1) |
| Male sex, n (%) | 459 (55) | - | 459 (55) |
| BMI <18.5 or ≥31, n (%) | 91 (11) | 2 (0) | 92 (11) |
| Charlson Comorbidity Index, n (%)  <2  ≥2 | 471 (56)  364 (44) | 1 (0) | 472 (56)  364 (44) |
| ASA classification, n (%)  I-II  III-IV | 636 (76)  188 (22) | 12 (1) | 644 (77)  192 (23) |
| ECOG performance score at primary diagnosis, n (%)  0-1  2-4 | 463 (55)  58 (7) | 315 (38) | 727 (87)  109 (13) |
| Preoperative serum CA 19-9, median (IQR) | 126 (31-480) | 277 (33) | 126 (30-480) |
| Preoperative bilirubin (µmol/L), median (IQR) | 24 (9-89) | 435 (52) | 24 (9-89) |
| Preoperative eGFR (ml/min/1,73 m^2^), n (%)  Normal (>90)  Mildly decreased (60-89)  Mildly to moderately decreased (45-59)  Moderately to severely decreased (30-45)  Severely decreased (<30) | 104 (12)  249 (30)  34 (4)  11 (1)  2 (0) | 436 (52) | 234 (28)  462 (55)  93 (11)  42 (5)  5 (1) |
| Preoperative anemia, n (%) | 198 (24) | 435 (52) | 441 (53) |
| Number of medicaments, n (%)  <5  ≥5 | 510 (61)  326 (39) | - | 510 (61)  326 (39) |
| Neoadjuvant chemotherapy, n (%) | 62 (7) | - | 62 (7) |
| Type of surgery, n (%)  Open  Laparoscopic  Robot | 755 (90)  73 (9)  6 (1) | 2 (0) | 757 (91)  73 (9)  6 (1) |
| Type resection, n (%)  Pancreatoduodenectomy  Distal pancreatectomy  Total pancreatectomy | 681 (81)  120 (14)  34 (4) | 1 (0) | 682 (82)  120 (14)  34 (4) |
| Location tumor, n (%)  Head  Body/tail | 709 (85)  127 (15) | - | 709 (85)  127 (15) |
| Vascular resection, n (%) | 227 (27) | 2 (0) | 228 (27) |
| Microscopic perineural invasion, n (%) | 635 (76) | 113 (14) | 726 (87) |
| Microscopic lymphovascular invasion, n (%) | 406 (49) | 227 (27) | 544 (65) |
| Tumor size in cm, mean ± SD | 3.2 ± 1.3 | 18 (2) | 3.2 ± 1.3 |
| Tumor differentiation, n (%)  Well/moderate  Poor | 505 (60)  232 (28) | 99 (12) | 574 (69)  262 (31) |
| Total number of resected lymph nodes, median (IQR) | 15 (10-21) | 17 (2) | 15 (10-21) |
| Number of positive lymph nodes, median (IQR) | 2 (0-4) | 6 (1) | 2 (0-4) |
| TNM stage 7^th^ AJCC edition, n (%)  Stage 1-2  Stage 3-4 | 75 (9)  748 (89) | 13 (2) | 77 (9)  759 (91) |
| Resection margin status, n (%)  R0 >1.0 mm  R1 ≤1.0 mm | 407 (49)  420 (50) | 9 (1) | 412 (49)  424 (51) |
| Major complications, n (%) | 241 (29) | - | *-* |
| Length of hospital stay (days), median (IQR) | 11 (8-17) | - | - |
| Adjuvant chemotherapy, n (%) | 500 (60) | 28 (3) | - |
| Reason no adjuvant chemotherapy, n (%)^~^  Per protocol  Disease progression  Toxicity  Poor overall performance status  Patients’ wish  Age  Death  Other | 16 (5)  28 (8)  -  90 (27)  83 (25)  18 (5)  50 (15)  7 (2) | 42 (13) | - |
| Type adjuvant chemotherapy, n (%)^#^  Gemcitabine monotherapy  FOLFIRINOX  Other | 474 (95)  6 (1)  13 (3) | 7 (1) | - |
| Number of cycli of adjuvant chemotherapy, median (IQR)^#^ | 2 (0-6) | 49 (10) | - |
| ≥80% of prescribed cycles completed, n (%)^#^ | 325 (7) | 49 (10) | - |
| Reason stop adjuvant chemotherapy, n (%)^#^  Per protocol  Disease progression  Toxicity  Poor overall performance status  Patients’ wish  Death  Unknown | 273 (55)  37 (7)  96 (19)  24 (5)  23 (5)  1 (0)  2 (0) | 45 (9) | - |
| 90-day mortality, n (%) | 49 (6) | - | - |
| Overall survival in months, median (IQR) | 19 (11-36) | - | - |
| Disease-free survival in months, median (IQR) | 14 (9-29) | 145 (17) | - |
| Recurrence, n (%) | 557 (67) | 145 (17) | - |
| Location recurrence, n (%)^^^  Local-only  Liver-only  Lung-only  Multiple sites  Other isolated distant site | 111 (20)  78 (14)  30 (5)  287 (52)  25 (4) | 20 (4) | - |
| Percentages may not sum to 100 because of rounding.  * Maximum diameter of the tumor  ^~^ Calculated in a subset of patients who did not start with adjuvant chemotherapy (336 patients)  ^#^ Calculated in a subset of patients who started with adjuvant chemotherapy (500 patients)  ^^^ Calculated in a subset of patients who developed recurrence of pancreatic cancer (557 patients)  SD, standard deviation; BMI, body mass index; ASA, American Society of Anesthesiologists; ECOG, Eastern Cooperative Oncology Group; IQR, interquartile range; AJCC, American Joint Committee on Cancer; FOLFIRINOX, 5-fluorouracil, leucovorin, irinotecan, oxaliplatin chemotherapy | | | |

| **Appendix Table 2. Univariate analysis of frailty characteristics of 71 patients aged ≥75 years receiving adjuvant chemotherapy and 119 patients aged ≥75 years not receiving adjuvant chemotherapy after imputation** | | | |
| --- | --- | --- | --- |
|  | **Adjuvant chemotherapy**  **N=71** | **No adjuvant chemotherapy**  **N=119** | **P-value^#^** |
| Polypharmacy, n (%) | 29 (41) | 59 (50) | 0.31 |
| Anemia, n (%) | 35 (50) | 67 (56) | 0.52 |
| Renal function, eGFR (ml/min/1.73 m^2^), n (%)  Normal (>90)  Mildly decreased (60-89)  Mildly to moderately decreased (45-59)  Moderately to severely decreased (30-45)  Severely decreased (<30) | 13 (18)  49 (68)  7 (10)  2 (3)  - | 26 (22)  72 (61)  11 (9)  8 (7)  3 (2) | 0.50 |
| CCI≥2, n (%) | 31 (44) | 81 (68) | 0.002 |
| BMI <18.5 or ≥30, n (%) | 3 (4) | 14 (12) | 0.13 |
| ASA score≥3, n (%) | 14 (20) | 49 (41) | 0.005 |
| Percentages may not sum to 100 because of rounding.  ^#^ The data were statistically analyzed between both groups with the chi-square test for categorical variables and with the Wilcoxon rank test for non-normally distributed continuous variables.  eGFR, estimated glomerular filtration rate; IQR, interquartile range; CACI, Charlson Comorbidity Index; BMI, body mass index; ASA, American Society of Anesthesiologists. | | | |

| **Appendix Table 3. Univariate analysis of 783 patients aged <80 years and 53 patients aged ≥80 years after resection for pancreatic cancer** | | | |
| --- | --- | --- | --- |
|  | **Age <80 years**  **N=783** | **Age ≥80 years**  **N=53** | **P-value^#^** |
| Male sex, n (%) | 431 (55) | 28 (53) | 0.86 |
| BMI <18.5 or ≥31, n (%) | 89 (11) | 2 (4) | 0.14 |
| Charlson Comorbidity Index, n (%)  <2  ≥2 | 464 (59)  319 (41) | 8 (15)  45 (85) | <0.001 |
| ASA classification, n (%)  I-II  III-IV | 608 (78)  175 (22) | 35 (67)  18 (33) | 0.10 |
| ECOG performance score at primary diagnosis, n (%)  0-1  2-4 | 685 (88)  98 (12) | 42 (79)  11 (21) | 0.11 |
| Preoperative serum log CA 19-9, median (IQR) | 122 (30-480) | 210 (31-833) | <0.01 |
| Preoperative bilirubin (µmol/L), median (IQR) | 24 (9-89) | 24 (9-84) | 0.99 |
| Preoperative eGFR (ml/min/1,73 m^2^), n (%)  Normal (>90)  Mildly decreased (60-89)  Mildly to moderately decreased (45-59)  Moderately to severely decreased (30-45)  Severely decreased (<30) | 226 (29)  426 (54)  88 (11)  39 (5)  5 (1) | 9 (16)  36 (67)  5 (9)  4 (7)  - | 0.24 |
| Preoperative anemia, n (%) | 409 (52) | 32 (60) | 0.37 |
| Number of medicaments, n (%)  <5  ≥5 | 484 (62)  299 (38) | 26 (49)  27 (51) | 0.09 |
| Neoadjuvant chemotherapy, n (%) | 60 (8) | 2 (4) | 0.42 |
| Method of surgery, n (%)  Open  Laparoscopic  Robot | 713 (91)  65 (8)  5 (1) | 44 (83)  8 (15)  1 (2) | 0.10 |
| Type resection, n (%)  Pancreatoduodenectomy  Distal pancreatectomy  Total pancreatectomy | 636 (81)  114 (15)  33 (4) | 46 (87)  6 (11)  1 (2) | 0.69 |
| Location tumor, n (%)  Head  Body/tail | 662 (85)  121 (15) | 47 (89)  6 (11) | 0.54 |
| Vascular resection, n (%) | 216 (28) | 12 (23) | 0.54 |
| Microscopic perineural invasion, n (%) | 683 (87) | 43 (82) | 0.37 |
| Microscopic lymphovascular invasion, n (%) | 512 (65) | 32 (60) | 0.48 |
| Tumor size in cm*, mean ± SD | 3.2 ± 1.3 | 3.2 ± 1.1 | 0.53 |
| Tumor differentiation, n (%)  Well/moderate  Poor | 439 (56)  244 (31) | 35 (66)  18 (34) | 0.77 |
| Total number of resected lymph nodes, median (IQR) | 15 (11-21) | 12 (9-15) | <0.001 |
| Number of positive lymph nodes, median (IQR) | 2 (0-4) | 2 (0-3) | 0.03 |
| TNM stage 7^th^ AJCC edition, n (%)  ≤ stage 2a  ≥ stage 2b | 74 (10)  709 (90) | 3 (6)  50 (94) | 0.47 |
| Resection margin status, n (%)  R0 >1.0 mm  R1 ≤1.0 mm | 387 (49)  396 (51) | 25 (47)  28 (53) | 0.87 |
| Percentages may not sum to 100 because of rounding.  ^a^ 2 missings; ^b^ 12 missings  ^#^ The data were statistically analyzed between both groups with the chi-square test for categorical variables and with the Fisher Exact Test when groups consist of less than 5 patients. A T test was used for normally distributed continues variables, and the Wilcoxon Rank test for non-normally distributed continues variables.  *Maximum diameter of the tumor  **Calculated in a subset of patients who started with adjuvant chemotherapy (495 patients aged <80 years vs. 5 patients aged ≥80 years)  SD, standard deviation; BMI, body mass index; ASA, American Society of Anesthesiologists; ECOG, Eastern Cooperative Oncology Group; IQR, interquartile range; AJCC, American Joint Committee on Cancer | | | |

| **Appendix Table 4. Multivariable logistic regression analysis to assess the impact of age ≥80 years on 90-day complication-related mortality, assessed for frailty** | | | |
| --- | --- | --- | --- |
|  | **90-day complication-related mortality** | | |
|  | **OR** | **95% CI** | **P value** |
| Age (≥80 vs. <80 years) | 2.00 | 0.81-4.96 | 0.14 |
| Charlson Comorbidity Index (n) | 1.21 | 0.99-1.48 | 0.07 |
| Number of medicaments (n) | 1.07 | 0.98-1.17 | 0.12 |
| Preoperative hemoglobin (mmol/L) | 0.92 | 0.69-1.22 | 0.56 |
| BMI (kg/m^2^) | 1.01 | 0.95-1.08 | 0.71 |
| Preoperative eGFR (ml/min/1.73 m^2^) | 0.99 | 0.98-1.02 | 0.68 |
| OR, odds ratio; 95% CI, 95% confidence interval; BMI, body mass index; eGFR, estimated glomerular filtration rate | | | |
